# Continuous Cropping Is Associated with Shifts in Strawberry Root-Associated Bacterial Communities and Predicted Carbon–Nitrogen Functional Profiles

**DOI:** 10.3390/biology15151229

**Published:** 2026-07-23

**Authors:** Ming Tao, Muhammad Umer, Rongrong You, Kangjian Song, Naureen Anwar, Muhammad Waseem, Hongcha Zhang, Huaming Lei, Qinxin Tao, Canting Ren, Shiping Jiang, Zhifang He, Xiaorou Huang, Sidra Ashraf, Xiande Duan, Jieming Feng, Mingzheng Duan

**Affiliations:** 1 Advanced Institute of Ecological Agriculture and Biodiversity on the Yunnan-Guizhou Plateau, Zhaotong University, Zhaotong 657000, China; 202315120111@stu.ztu.edu.cn (M.T.); 202215040109@stu.ztu.edu.cn (R.Y.); 202315120109@stu.ztu.edu.cn (K.S.); 202315120116@stu.ztu.edu.cn (H.Z.); 202315120140@stu.ztu.edu.cn (H.L.); 202315120112@stu.ztu.edu.cn (Q.T.); 202515120105@stu.ztu.edu.cn (C.R.); shipingjiang2004@163.com (S.J.); 202215040106@stu.ztu.edu.cn (Z.H.); 202315040204@stu.ztu.edu.cn (X.H.); yl2024030029@ztu.edu.cn (X.D.); 2 School of Breeding and Multiplication (Sanya Institute of Breeding and Multiplication), College of Tropical Agriculture and Forestry, Hainan University, Sanya 572025, China; umer.hzau@outlook.com (M.U.); m.waseem.botanist@gmail.com (M.W.); 3 Department of Biological Sciences, Virtual University of Pakistan, Raiwind Road Campus, Lahore 54000, Punjab, Pakistan; naureen.anwar@vu.edu.pk; 4 Department of Biochemistry, University of Agriculture Faisalabad, Faisalabad 38000, Punjab, Pakistan; sidra.best09@gmail.com

**Keywords:** co-occurrence network, continuous cropping, microbial community structure, FAPROTAX, predicted carbon–nitrogen functional profiles

## Abstract

Long-term cultivation of strawberries can alter plant-associated microbial communities; however, the responses of root-associated bacteria within the combined rhizoplane and endophytic community remain insufficiently characterized. This study compared bacterial community composition, co-occurrence patterns, and predicted ecological functions in root-associated bacteria from short-term (4 months; 4 mon) and long-term (16 months; 16 mon) cultivation of Dandong 99 strawberry plants. Because the two groups differed simultaneously in soil cultivation history, plant age, and root developmental stage, all observed differences are interpreted as correlates of cultivation duration rather than as effects of continuous cropping per season. The 6 composite root samples were analyzed using Illumina NovaSeq sequencing of the bacterial 16S rRNA V4 region, followed by ASV-based diversity analysis, taxonomic profiling, LEfSe, FastSpar-based co-occurrence network analysis, and FAPROTAX functional prediction. Across all samples, 264,350 high-quality sequences and 11,442 ASVs were obtained. Long-term cultivation was associated with lower observed ASV richness and a marked shift in community composition (PERMANOVA R^2^ = 0.935; ANOSIM R = 1). At the phylum level, Actinobacteria declined significantly (30.50% to 12.85%), while Proteobacteria and Bacteroidota were enriched. At the genus level, *Streptomyces*, *Steroidobacter*, *Bradyrhizobium*, and *unidentified Rhizobiaceae* decreased significantly. Network analysis identified *Sphingobium*, *Bradyrhizobium*, and *Steroidobacter* as highly connected genera by degree centrality, with the latter two also showing reduced relative abundance. Functional prediction indicated significantly lower predicted chemoheterotrophy, aerobic chemoheterotrophy, nitrogen fixation, nitrate reduction, and chitinolysis, alongside higher predicted methylotrophy, methanol oxidation, ureolysis, and aromatic compound degradation. These findings provide genus-level evidence that cultivation duration is associated with compositional and predicted functional shifts in strawberry root-associated bacteria. The depleted Actinobacteria and *Bradyrhizobium* partially align with microbial signatures of continuous cropping obstacles, warranting future validation in age-controlled, temporally replicated studies.

## Simple Summary

This study investigated how cultivation duration influences root-associated bacterial communities in strawberry (cv. Dandong 99), focusing on combined rhizoplane and endophytic compartments. Using 16S rRNA (V4) amplicon sequencing, bacterial diversity, composition, co-occurrence networks, and predicted functions were compared between short-term (4 months) and long-term (16 months) cultivation. Long-term cultivation significantly reduced bacterial richness and altered community structure. Notably, Actinobacteria declined, while Proteobacteria and Bacteroidota increased. Several genera, including *Streptomyces* and *Bradyrhizobium*, decreased in abundance, despite some remaining central in network connectivity. Functional predictions suggested reduced capacities for chemoheterotrophy, nitrogen fixation, and chitinolysis, alongside increased methylotrophy and aromatic compound degradation. Overall, the results indicate that extended cultivation duration is associated with shifts in both the composition and potential function of root-associated bacterial communities, partially resembling microbial patterns linked to continuous cropping stress.

## 1. Introduction

Strawberry (*Fragaria* × *ananassa* Duch.) is an important fruit crop valued worldwide for its large size, bright color, rich flavor, and unique taste [1]. To alleviate land scarcity and reduce production costs, a cultivation pattern of single planting with multiple years of harvest is becoming increasingly common in strawberry production [1]. However, as the planting year extends, even under standard management, strawberries generally exhibit declining disease resistance, aggravated soil-borne diseases, and continuous decreases in yield and quality [2], which in turn lead to excessive application of pesticides and fertilizers, posing food safety risks and constraining the sustainable development of the industry [3,4]. This comprehensive decline observed when the same crop is consecutively cultivated on the same land is termed the continuous cropping obstacle [5]. Existing studies have confirmed that in long-term strawberry cropping systems, the accumulation of phenolic acid autotoxins [2], deterioration of soil physicochemical properties [5], and microbial community imbalance [6] are the 3 core driving factors of continuous cropping obstacles. For example, under long-term continuous cropping conditions, reduced daidzein exudation from soybean roots diminishes *Bradyrhizobium* recruitment capacity, thereby impairing biological nitrogen fixation efficiency, leading to insufficient plant nitrogen nutrition and decreased yield [7]. A comparative study of continuous-cropping-tolerant and continuous-cropping-sensitive soybean cultivars further demonstrated that tolerant cultivars sustain higher *Bradyrhizobium* abundance in the rhizosphere and a more stable microbial network structure, thereby alleviating the obstacles posed by continuous cropping [8].

The rhizosphere, the narrow zone of soil directly influenced by root activity, is a hotspot for studying soil–plant–microbe interactions [9]. It has been clearly demonstrated that long-term continuous cropping significantly alters the rhizosphere microbial community structure of strawberry, characterized by reduced community diversity, a shift from a bacterial-dominated to a fungal-dominated type, accompanied by the enrichment of soil-borne pathogens such as *Fusarium oxysporum* [10] and the depletion of beneficial microorganisms such as *Bacillus* spp. and *Trichoderma* spp. [11,12]. However, plant-associated microorganisms are not confined to the rhizosphere. From the rhizoplane to the endosphere, there exist bacterial communities that are in closer physical contact and more direct physiological association with the host, collectively referred to as root-associated bacteria [13]. Unlike the open and environmentally buffered rhizosphere microbiota, root-associated bacteria reside at the frontline of plant–microbe interactions and are considered to play pivotal roles in regulating host growth [14], nutrient uptake, stress defense [15], and even disease occurrence [16]. It has been reported that *Azospirillum brasilense* can stably colonize strawberry roots and exert significant growth-promoting effects [16,17]. To date, studies on strawberry root-associated bacteria have primarily focused on the isolation [18], identification [19], and verification of growth-promoting and biocontrol functions of culturable endophytes in healthy plants [20], whereas their community succession, interspecific interactions, and potential ecological functional shifts under long-term planting conditions remain poorly understood. Only a limited number of studies have investigated the diversity and functional prediction of soil microbial communities under continuous strawberry cropping [6]. In contrast, the effects of root-associated bacteria, which maintain intimate physical contact with host plants, on strawberry growth and health remain largely unreported.

To dissect these differences in depth, this study adopted a combined technical approach of community composition co-occurrence network and functional prediction. First, 16S rRNA gene amplicon sequencing, as a mature culture-independent and high-throughput technique, can effectively overcome the challenges of low biomass of root-associated bacteria and high host genome interference [21], enabling precise elucidation of community structure and diversity [22]. For instance, Zhang et al. [22] employed 16S rRNA sequencing to characterize the structure and diversity of strawberry root endophytic bacteria under different cultivation substrates, and similarly [23]. Second, co-occurrence network analysis can mine coexisting or mutually exclusive relationships among microbial taxa from massive sequencing data, identify core hub taxa that maintain community stability, and reveal potential network disassembly and reorganisation under long-term planting stress [6,12]. For example, co-occurrence network analysis has been applied to explore bacterial networks and coexistence relationships in the lettuce rhizosphere microbiome [23], and shifts in microbial community network structure have been observed in the strawberry microbiome after *Fusarium* wilt infection [24]. Finally, the FAPROTAX functional prediction tool can link microbial community composition data to potential ecological functions, predict core ecological functions of microbial communities, clarify the patterns of functional changes driven by continuous cropping stress, and infer the potential ecological functional spectra such as carbon, nitrogen, and sulfur cycling [25,26], thereby compensating for the limitation that amplicon sequencing cannot directly predict function. The integrated application of these three approaches can systematically address the progressive scientific questions of who is present, how they interact, and what they might do.

Accordingly, we propose the following exploratory hypothesis: under the composite stress of long-term strawberry cultivation, the root-associated bacterial community will exhibit dysbiosis characterized by the loss of beneficial bacteria and the enrichment of potential pathogens, with a simplified and destabilized network topology, and attenuated functional potential for nutrient cycling. These features partially coincide with the classical microbial signatures of continuous cropping obstacles. The aims of the present study are (1) to elucidate the effects of extended planting duration on the diversity, composition, and potential functions of strawberry root-associated bacterial communities; (2) to identify key bacterial taxa significantly associated with planting duration. We anticipate that our study will provide critical hypotheses and target microbial groups for future experiments that strictly control plant age to isolate and generate hypotheses for future studies with expanded temporal replication and environmental metadata.

## 2. Materials and Methods

### 2.1. Experimental Design

This study used a single-time-point field comparison in May 2023. Roots of Dandong 99 strawberries that had been continuously cropped for 16 mon and exhibited obvious continuous-cropping obstacles were selected as the long-term planting group (SG). Roots of healthy Dandong 99 strawberries were newly planted for 4 mon; during the same period, the control group (CK) served as a short-term planting group. The experiment for each group comprised 3 ridges, each serving as a plot of approximately 4.5 m^2^ (7.5 m × 0.6 m), with plants spaced at 25 cm in double rows. At sampling, plants from the outer row of each ridge were excluded to avoid edge effects. It is a defining feature of this design that the SG group differs from the CK group in both short-term and long-term (4 vs. 16 mon) cultivations and in root system developmental stage. Accordingly, any measured differences between these 2 groups reflect the combined response to these closely related factors and cannot be attributed to continuous cropping solely per season.

The experimental soil was sandy brown soil (local agricultural brown earth) collected from a greenhouse field in Zhaotong, Yunnan and selected soil physicochemical properties were measured at the beginning of the experiment: bulk density, 1.06 g/cm^3^ (core ring method) [27]; alkali-hydrolyzable nitrogen, 62 mg/kg (alkaline hydrolysis diffusion method) [28]; available phosphorus, 16 mg/kg (Olsen extraction-molybdenum blue colorimetric method) [29]; Available potassium, 42.16 mg/kg (NH_4_OAc extraction method) [30]; pH, 7.6 (pH meter method); and electrical conductivity (EC), 0.128 mS/cm (bench-top conductivity meter method). The cultivation conditions were natural light and ambient greenhouse temperature, and conventional irrigation was applied throughout the experiment. During the experiment, no fertilization or plant protection measures were used to minimize external interference. Although this design cannot disengage the effects of plant age from those of continuous cropping, the comparison between the SG and CK groups captures the integrated shift in the root-associated bacterial community that accompanies the transition from a healthy young root system to a declined older one precisely. This scenario defines continuous-cropping obstacles in strawberry production. The present study therefore focused on characterizing this combined microbial response and on identifying bacterial taxa potentially indicative of the decline in plant performance under prolonged cultivation.

### 2.2. Sample Collection

During sampling, whole plants were carefully uprooted, and their roots were gently shaken to remove bulk soil. Soil remaining on the root surface was removed with a sterile brush, followed by repeated rinsing with sterile ddH_2_O; however, a standard surface-sterilization procedure was not applied. Therefore, the bacterial community analyzed in this study represents the true colonized bacterial communities on the root surface (rhizoplane) and within root tissues (endosphere), and these communities are hereafter referred to as the root-associated bacterial community.

The 3 plants with uniform growth were randomly selected from each group. Given that fine roots frequently penetrate deep into the soil and are prone to breakage during sampling, making it unfeasible to maintain consistent fine root abundance across all samples, and that variations in root function and developmental stage can readily obscure the diversity characteristics of target root-associated microbial communities, sampling was focused on the thicker primary roots of each plant to maximally ensure that the collected samples capture the effects of planting duration on functional primary roots of strawberry. Cleaned root samples were composited into a single biological replicate, and each group included 3 composite biological replicates, yielding a total of 6 samples. Therefore, each replicate represented a composite root sample rather than an individual plant, and the collected samples were immediately frozen in liquid nitrogen and then stored at −80 °C for subsequent analyses.

### 2.3. DNA Extraction, PCR Amplification, and High-Throughput Sequencing

Genomic DNA was extracted from approximately 0.5 g of root tissue per composite sample using the CTAB method, and DNA purity and concentration were assessed using a NanoDrop spectrophotometer (Thermo Fisher Scientific Inc., Wilmington, DE, USA) and by agarose gel electrophoresis [31]. The DNA was then diluted to 1 ng/μL with sterile ddH_2_O for use as the PCR template.

The V4 hypervariable region of the bacterial 16S rRNA gene was amplified using the barcode-labeled 515f (5′-GTGCCAGCMGCCGCGGTAA-3′) and 806r (5′-GGACTACHVGGGTWTCTAAT-3′) primers, with unique barcode sequences incorporated into the forward primer. PCR was performed using Phusion^®^ High-Fidelity PCR Master Mix with GC Buffer and high-fidelity DNA polymerase (New England Biolabs, Inc., Ipswich, MA, USA) to ensure amplification efficiency and accuracy. PCR reactions were carried out in a total volume of 30 μL, containing 15 μL of the master mix, 0.2 μM of each primer, and approximately 10 ng of template DNA. The thermal cycling conditions were as follows: initial denaturation at 98 °C for 1 min; followed by 30 cycles of denaturation at 98 °C for 10 s, annealing at 50 °C for 30 s, and extension at 72 °C for 30 s; with a final extension at 72 °C for 5 min. Negative controls (PCR-grade sterile ddH_2_O instead of a DNA template) were included in both the DNA extraction and PCR amplification steps to monitor potential reagent and environmental contamination.

PCR products were visualized on a 2% agarose gel, and the target bands were purified using a gel extraction kit (Qiagen GmbH, Hilden, NRW, Germany). Following purification and quantification, sequencing libraries were constructed using the TruSeq^®^ DNA PCR-Free Sample Preparation Kit(Illumina, Inc., San Diego, CA, USA). Library concentration and quality were evaluated using Qubit and qPCR. Qualified libraries were then subjected to paired-end sequencing (2 × 250 bp) on the Illumina NovaSeq 6000 platform. Then, the high-throughput sequencing was performed by Wuhan Metware Biotechnology Co., Ltd., Wuhan, China.

### 2.4. Bioinformatics and Statistical Analysis

Raw sequencing data were first subjected to quality control, and raw paired-end reads were demultiplexed based on barcode and primer sequences. Primers and barcodes were trimmed, and low-quality reads were filtered using fastp (v0.22.0, https://github.com/OpenGene/fastp; accessed on 20 January 2026) with the following criteria: automatic adapter detection and removal; discarding reads with ≥15 N bases or with >50% low-quality bases (Q ≤ 20); sliding window trimming with an average quality threshold of 20 over a 4-base window; removal of polyG tails; and exclusion of reads < 150 bp [32]. Paired-end reads were assembled using FLASH (v1.2.11; http://ccb.jhu.edu/software/FLASH; accessed on 20 January 2026)to generate clean tags [33]. Subsequently, chimera sequences were detected and removed by aligning against the SILVA 138.1 reference database using vsearch (v2.22.1, https://github.com/torognes/vsearch; accessed on 20 January 2026), resulting in final effective tags for downstream analysis [34].

Amplicon sequence variants (ASVs) were generated by sequence denoising using the Deblur algorithm (v1.1.1) implemented in QIIME 2 (v2023.2) [35], which clusters clean tags into ASVs at 100% sequence similarity. We present the detailed statistics of ASVs from amplicon sequencing in Table S1. Species annotation was performed using representative ASVs with the Mothur (v1.48) implementation of the RDP classifier method and the SILVA (v138; http://www.arb-silva.de; accessed on 15 March 2026) SSU rRNA database [36]. A confidence threshold of 0.8–1.0 was used, and community composition was summarized at the phylum, class, order, family, genus, and species levels. Multiple sequence alignment of ASV representative sequences was conducted using MAFFT (v7.520, https://mafft.cbrc.jp/alignment/software; accessed on 22 March 2026) to construct a phylogenetic tree [37]. All samples were rarefied to the minimum sequencing depth to normalize data; all subsequent alpha and beta diversity analyses were based on this rarefied table. We present the detailed taxonomic information of amplicon sequencing in Table S2.

For Alpha diversity analysis, QIIME2 and R (v4.2.0) with the phyloseq (v1.40.0) and vegan (v2.6-2) packages were used to calculate alpha diversity indices [38], including Observed ASVs, Shannon index, Simpson, ACE, and Good’s coverage index, and differences between treatment groups were assessed using *t*-tests. For Beta diversity analysis, unifrac distances were computed using the phyloseq package. Principal component analysis (PCA) was performed with the stats package in R. Principal coordinates analysis (PCoA) and non-metric multidimensional scaling (NMDS) were performed based on Unifrac distances using the phyloseq package (v1.40.0) within R (v4.2.0) to evaluate differences in community structure. The ordinate function in the phyloseq package (v1.40.0) within R (v4.2.0), with Bray–Curtis dissimilarity calculated from rarefied ASV abundance data, was used. NMDS plots were generated using ggplot2 (v3.4.0). Differences in beta diversity among groups were evaluated using PERMANOVA and analysis of similarities (ANOSIM), both with 999 permutations. PERMANOVA was implemented with the adonis function in vegan package (v2.6-4) based on R software (v4.2.0). Pairwise comparisons were performed using *t*-tests and Wilcoxon tests as appropriate. Two-group comparisons of alpha diversity indices and differential genus abundances were conducted using the *t*-test function in R’s base stats package. Before applying *t*-tests, normality was assessed via the Shapiro–Wilk test and homogeneity of variances was evaluated using Levene’s test. For non-normal or heteroscedastic datasets, the Wilcoxon rank-sum test was used instead, implemented via the wilcox.test function in R’s base stats package.

Linear discriminant analysis effect size (LEfSe, v1.1.2) was applied to identify biomarker taxa with significant differences between groups [39]. Briefly, the LEfSe pipeline first performed a Kruskal–Wallis test, followed by pairwise Wilcoxon tests, and discriminative taxa were selected using a logarithmic LDA score threshold of 4.0. Additionally, Metastats analysis was performed using Mothur (version 1.48.0) to conduct permutation tests at each taxonomic level, with *p*-values corrected for multiple testing using the Benjamini–Hochberg false discovery rate (FDR) to obtain q-values. Taxa with significant differences were also identified using *t*-tests in R (v4.2.0).

Co-occurrence networks were constructed based on the relative abundance of the top 100 most abundant bacterial genera. Pairwise correlations were computed using FastSpar (v1.0.0), a reimplementation of the SparCC algorithm suitable for compositional data [40]. Edges were retained if the absolute correlation coefficient was >0.8; self-loops and edges connecting nodes with a mean relative abundance <0.005% were removed, and network visualization along with property calculation were performed in R [41].

For functional prediction, ASVs were annotated using the FAPROTAX database (http://www.loucalab.com/archive/FAPROTAX; accessed on 22 March 2026), which maps prokaryotic taxa to manually curated metabolic and ecological functions. Differences in the relative abundance of functional groups were analyzed between treatments using *t*-tests. To account for multiple testing across functional categories, Benjamini–Hochberg false discovery rate (FDR) correction was applied (Q < 0.05), and both raw *p*-values and FDR-adjusted Q-values are reported in the results. All statistical analyses and visualizations were performed in R (v4.2.0) using the ggplot2 (v3.4.0) and vegan packages unless otherwise stated.

To investigate the association between bacterial community composition and predicted functional potential, Pearson correlation coefficients were calculated between the relative abundances of the top 20 dominant genera and the top 20 functional categories from FAPROTAX annotation. Hierarchical clustering was applied to both the taxonomic (rows) and functional (columns) dimensions to group entries with similar correlation patterns [42].

The correlation matrix was visualized as a heatmap, with a color gradient indicating the direction and magnitude of the correlation (red for positive and green for negative correlation). Statistical significance of each pairwise correlation was denoted by asterisks: *, **, and *** indicated *p*-values < 0.05, <0.01, and <0.001, respectively. All correlation analyses and visualization were performed on the Metware Cloud platform (https://cloud.metware.cn; accessed on 2 April 2026).

## 3. Results

### 3.1. Diversity of Root-Associated Bacterial Communities Under Different Cultivation Durations

To characterize the diversity and community composition of root-associated bacteria in long-term (SG) and short-term (CK) strawberry cultivation, high-throughput sequencing of the 16S rRNA V4 region was performed using the Illumina platform. A total of 264,350 high-quality non-chimeric sequences were obtained across 6 samples, with a mean length of 376–380 bp and a Q30 score above 94.9% for all samples (Table S4). Each sample yielded an average of 2352 ASVs (range: 2070–3048) (Table S4).

**Figure 1:**
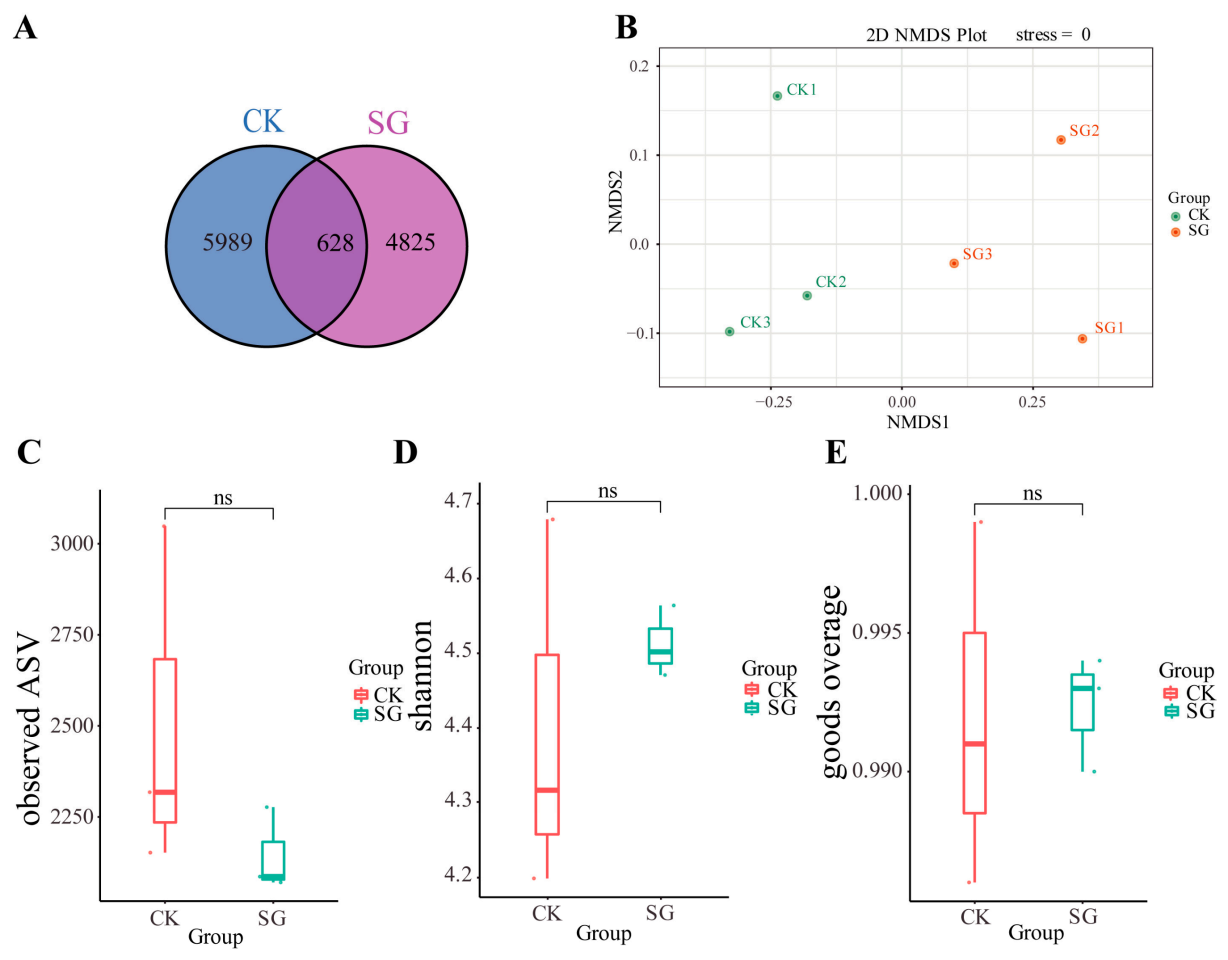
Diversity analysis of root-associated bacterial communities in strawberry under different cultivation durations (**A**) Venn diagram of amplicon sequence variants (ASVs) (SG) and control (CK) groups. (**B**) Non-metric multidimensional scaling (NMDS) plot of community structure based on ASV abundance (*n* = 3). Each point represents an individual sample. The distance between any two points corresponds to their degree of dissimilarity. (**C**) Observed ASVs (richness). The central horizontal line in each box plot denotes the median. (**D**) Shannon diversity index. (**E**) Good’s coverage index (*n* = 3). SG: long-term planting group; CK: short-term planting group; ASV: amplicon sequence variant; NMDS: non-metric multidimensional scaling.

Venn analysis of ASVs across groups (Figure 1A) identified 11,442 total ASVs, of which only 628 (5.49%) were shared across all 6 samples. The CK group contained 5989 unique ASVs (52.34%), while the SG group contained 4825 (42.17%). This pattern demonstrates a clear compositional differentiation between the two cultivation duration groups, with a limited proportion of ASVs universally shared across all replicates. This indicates a low proportion of core bacterial taxa and a high abundance of group-specific bacterial communities.

NMDS analysis based on Bray–Curtis dissimilarity of ASV relative abundance (Figure 1B) showed clear separation between the CK and SG groups along the first axis (Stress = 0). PERMANOVA confirmed that the cultivation duration explained 93.5% of the total compositional variation (R^2^ = 0.935, *p* = 0.1); ANOSIM further supported this separation (R = 1, *p* = 0.1). The *p*-values did not reach the conventional threshold of 0.05, which is attributable to the limited statistical power associated with 3 replicates per group; with *n* = 3, the minimum attainable *p*-value in a permutation-based test is 0.1. Nevertheless, the very high R^2^ and ANOSIM R values indicate a substantial differentiation in community structure between the two cultivation duration groups.

The CK group had a higher average number of ASVs (2506) than the SG group (2144) (Figure 1C), but this difference was not statistically significant (Wilcoxon test, *p* = 0.2). Consistent with this trend, the Chao1 richness estimator was also higher in the CK (2542.1 ± 514.2) than in the SG group (2180.7 ± 102.2) (Table S4), whereas the Shannon diversity index did not differ significantly between groups (CK: 4.40; SG: 4.51; Wilcoxon test, *p* = 0.7) (Figure 1D). The Simpson diversity index (1-D) was slightly higher in the SG (0.955 ± 0.003) than in the CK (0.922 ± 0.017) (Table S4), but this difference did not reach statistical significance (Wilcoxon test, *p* = 0.1). Good’s coverage values were greater than 0.98 for all samples (Figure 1E), indicating sufficient sequencing depth to represent bacterial community composition reliably. Taken together, these alpha diversity metrics suggest a trend toward lower taxonomic richness (Observed ASVs and Chao1) in the long-term cultivation group, while overall diversity (Shannon) and evenness (Simpson) remained comparable between groups.

### 3.2. Taxonomic Composition of Root-Associated Bacterial Communities Under Different Cultivation Durations

**Figure 2:**
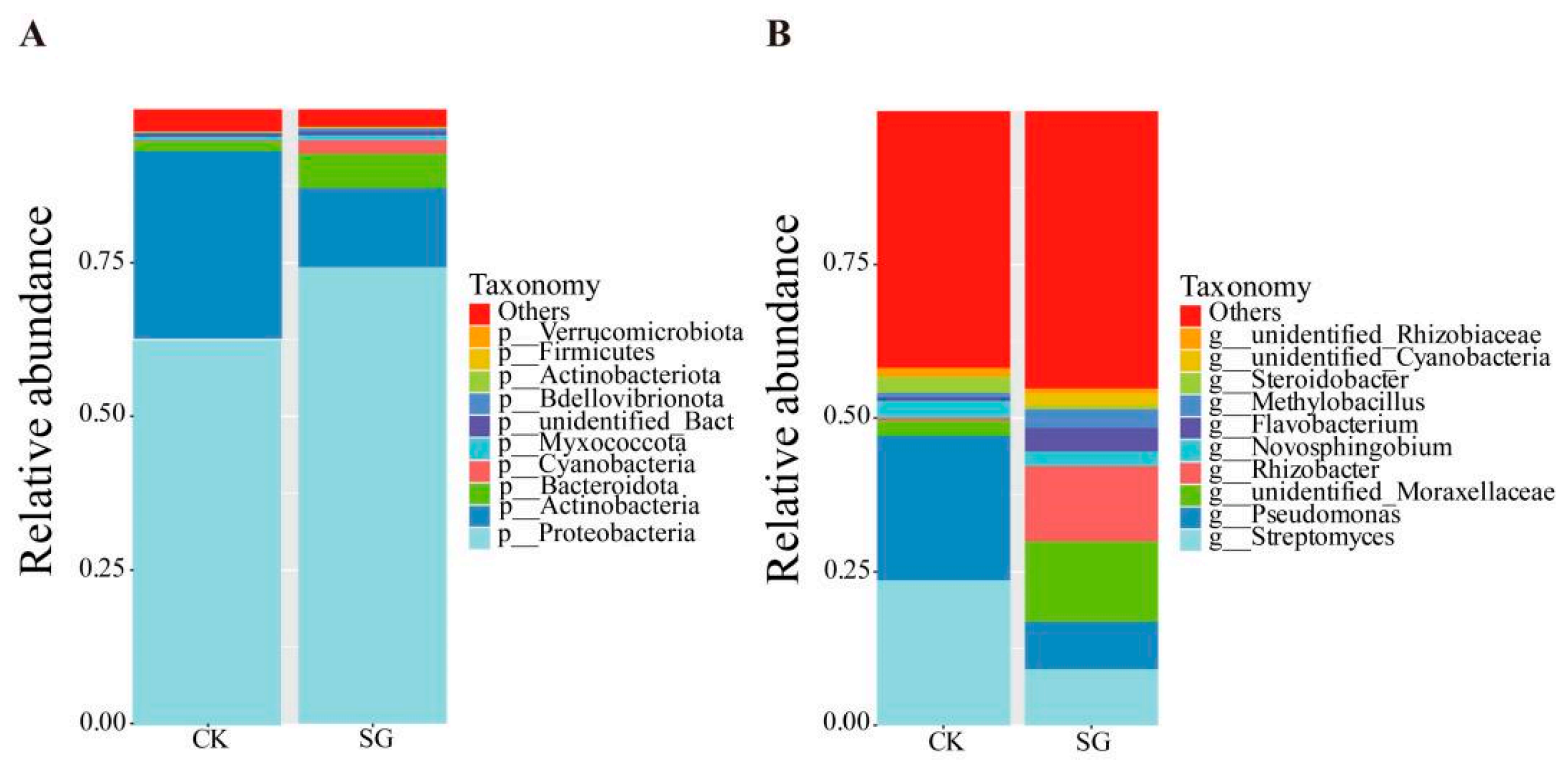
Associations between long-term cultivation duration and the community composition of strawberry root-associated bacteria. (**A**) Relative abundance of bacterial phyla in the long-term planting group (SG) and short-term planting group (CK). (**B**) Relative abundance of bacterial genera in the SG and CK groups.

**Figure 3:**
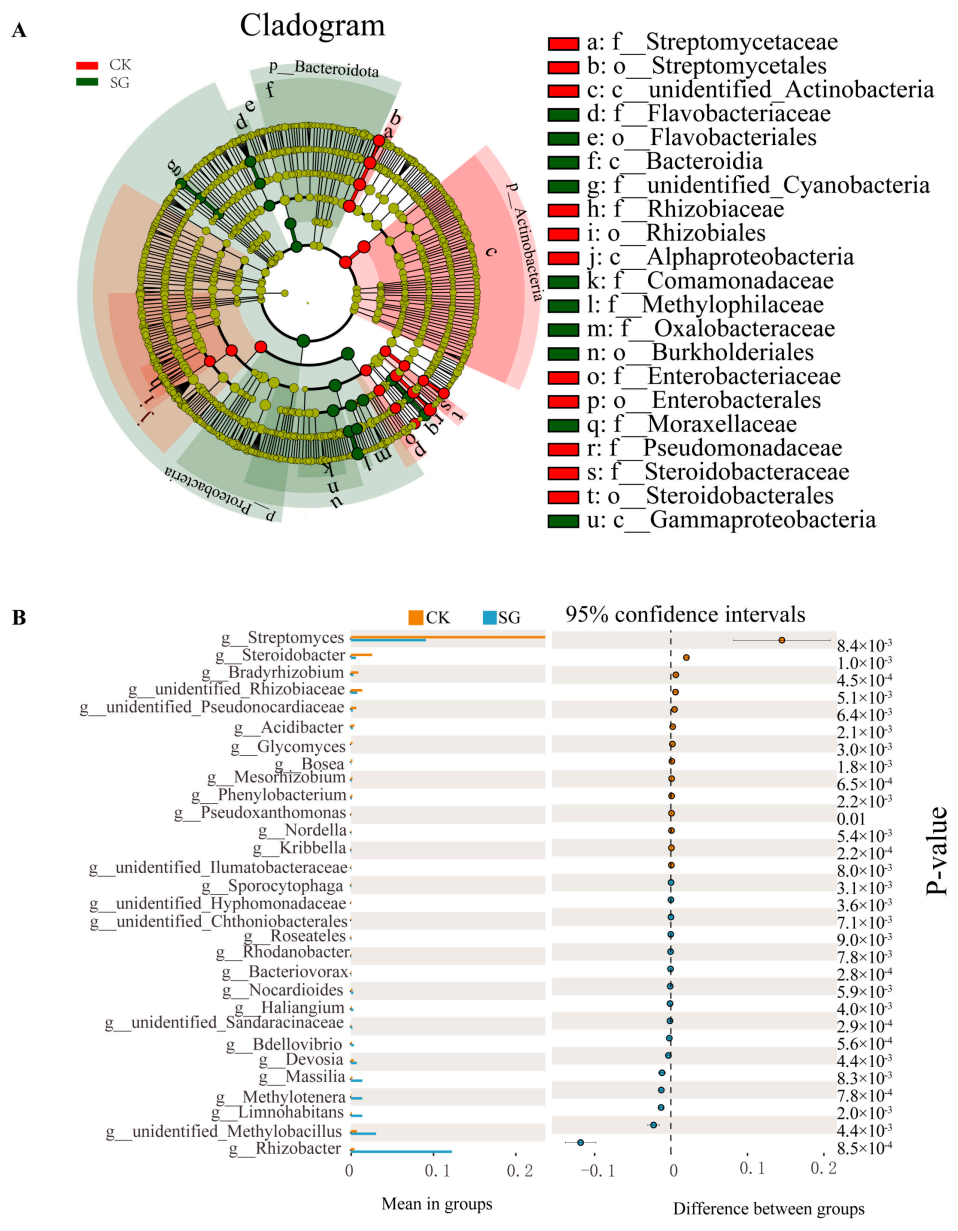
Analysis of significant differences in strawberry root-associated bacterial communities between treatments. (**A**) Linear discriminant analysis effect size (LEfSe) cladogram based on amplicon sequence variants (ASVs). The cladogram displays taxonomic levels from the phylum to the genus outward from the center. Each node represents a taxon, with node size proportional to relative abundance. Taxa without significant differences are shown in yellow, while biomarker taxa are colored according to the group in which they are enriched (red or green). Taxon names corresponding to labeled nodes are listed in the legend. (**B**) *t*-test analysis of differential genera. The left panel shows the mean relative abundance of genera with significant inter-group differences. The right panel shows the corresponding 95% confidence intervals (CIs) for mean differences. The center of each circle represents the mean difference, and the circle color indicates the group with higher abundance. The rightmost column reports the *p*-values for each genus. LEfSe: linear discriminant analysis effect size.

Analysis of bacterial community composition showed exponential differences between the CK and SG groups. At the phylum level (Figure 2A), Proteobacteria was the dominant phylum in both groups, with a relative abundance of 62.65% ± 5.13% in the CK group and 74.27% ± 0.70% in the SG group (*t*-test, *p* = 0.044) (Table S5). Actinobacteria, however, were significantly enriched in the CK group (30.50% ± 3.62%) compared to the SG group (12.85% ± 0.96%; *t*-test, *p* = 0.010) (Table S5). In contrast, Bacteroidota was significantly enriched in the SG group (5.68% ± 1.39%) relative to the CK group (1.64% ± 0.20%; *t*-test, *p* = 0.035) (Table S5). Given the compositional nature of relative abundance data, proportional shifts in dominant phyla may partly reflect redistribution rather than absolute changes in abundance. Linear discriminant analysis effect size (LEfSe, LDA score > 4.0) identified Actinobacteria as a biomarker phylum discriminative for the CK group, and Proteobacteria and Bacteroidota as discriminative for the SG group (Figure 3A). Given the limited sample size (*n* = 3 composite replicates per group), LEfSe results should be interpreted with caution, as the minimum attainable *p*-value in a Kruskal–Wallis test with 3 replicates per group is 0.0495; the biomarkers identified by LEfSe are only regarded as exploratory results for screening potential differential taxa, not as confirmatory conclusions.

At the genus level (Figure 2B), the enrichment abundance of microbial taxa differed substantially between groups. In the CK group, the dominant genera were *Streptomyces* (23.62% ± 4.66%), *Pseudomonas* (23.46% ± 0.08%), *Novosphingobium* (2.80% ± 0.17%), *Steroidobacter* (2.59%), and *unidentified_Moraxellaceae* (2.52% ± 0.26%). In contrast, the SG group was dominated by *unidentified_Moraxellaceae* (13.10% ± 2.04%), *Rhizobacter* (12.26% ± 0.88%), *Streptomyces* (9.09% ± 0.77%), *Pseudomonas* (7.73% ± 3.57%), and *Flavobacterium* (3.76% ± 1.41%). *t*-test analysis (Figure 3B) showed that continuous cropping significantly decreased the relative abundances of *Streptomyces* (from 23.6% to 9.1%, *p* = 0.008) (Table S6), *Steroidobacter* (from 2.6% to 0.6%, *p* = 0.008) (Table S6), *Bradyrhizobium* (from 0.9% to 0.3%, *p* = 0.0004) (Table S6), and *unidentified Rhizobiaceae* (from 1.4% to 0.8%, *p* = 0.005) (Table S6). *t*-test showed that the relative abundances of 4 genera were significantly lower in the SG group compared with the CK group: *Streptomyces* (*p* = 0.008) (Table S6), *Steroidobacter* (*p* = 0.008) (Table S6), *Bradyrhizobium* (*p* = 0.0004) (Table S6), and *unidentified_Rhizobiaceae* (*p* = 0.005) (Figure 3B) (Table S6). Among these, *Bradyrhizobium* was not individually displayed in the top 10 genera bar chart due to its low relative abundance, but its significant decline was robustly supported by differential statistical analysis. These results indicate that cultivation duration was associated with a marked decline in several taxa that were abundant in the CK group.

### 3.3. Co-Occurrence Network Analysis of Root-Associated Bacterial Genera

**Figure 4:**
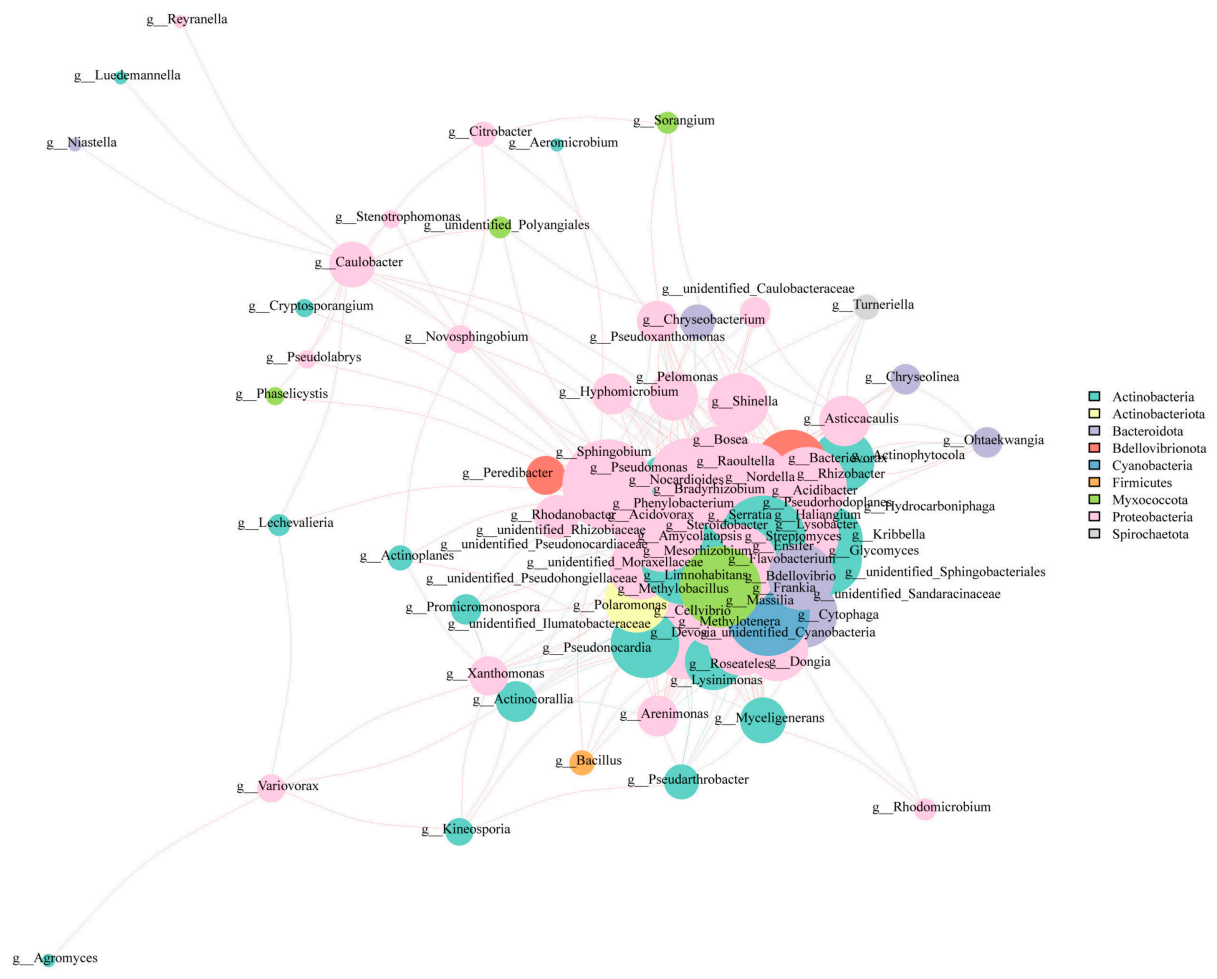
Correlation-based co-occurrence network of strawberry root-associated bacterial genera. Each node represents a genus, with node size proportional to degree (number of connections). Node colors correspond to phylum-level taxonomy. Edge thickness reflects the absolute value of the correlation coefficient, and edge color indicates correlation direction (red: positive; blue: negative). Because this network is based on correlation analysis, it reflects co-occurrence patterns rather than direct ecological interactions or community stability.

To further explore co-occurrence patterns among root-endophytic bacterial genera, a correlation-based co-occurrence network was constructed using FastSpar (v1.0.0) (Figure 4; Table S3). After filtering (correlation coefficient > 0.8), this network contained 26 key genera with an average degree of 25.74 and a clustering coefficient of 0.73 (Table S3). Among them, 3 genera (*Sphingobium* (Degree = 56), *Bradyrhizobium* (Degree = 54), and *Steroidobacter* (Degree = 52) exhibited the highest degree centrality, indicating that they occupied central positions in terms of direct co-occurrence associations. Notably, *Bradyrhizobium* and *Steroidobacter* were also identified in the previous *t*-test analysis (Figure 3B) as significantly lower in relative abundance in the SG group (*p* < 0.01). These results indicate that cultivation duration was associated with changes in the abundance of highly connected genera, suggesting altered co-occurrence patterns in the root-associated bacterial community. However, it should be emphasized that correlation-based networks do not distinguish between direct ecological interactions and indirect co-variation driven by shared environmental preferences. Therefore, these results should be viewed as descriptive hypotheses regarding potential microbial associations rather than as evidence of altered ecological interactions.

### 3.4. FAPROTAX-Predicted Functional Profiles of Root-Associated Bacterial Communities

**Figure 5:**
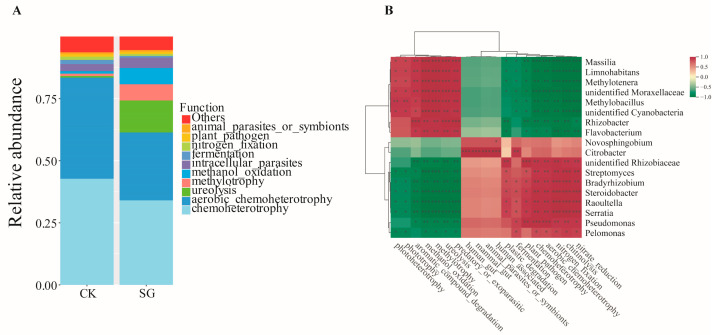
FAPROTAX-predicted functional profiles and genus-function correlation analysis of strawberry root-associated bacterial communities. (**A**) Relative abundance of FAPROTAX-predicted functional categories in the continuous-cropping group (SG) and control group (CK). These functions were inferred from 16S rRNA amplicon data and should be interpreted as prediction-based functional profiles rather than direct measurements of in situ metabolic activity. (**B**) Correlation heatmap of dominant bacterial genera (rows) and major predicted functional categories (columns) based on Pearson correlation coefficients, with hierarchical clustering performed on both axes. The color gradient ranges from red (positive correlation) to green (negative correlation), representing the direction and magnitude of the correlation. Statistical significance is marked as follows: *, *p* < 0.05; **, *p* < 0.01; ***, *p* < 0.001.

To explore the potential functional implications of the observed taxonomic differences, FAPROTAX-based functional prediction was performed using ASV profiles (Figure 5). The predicted functional profiles, together with *t*-tests with Benjamini–Hochberg false discovery rate (FDR) correction, revealed significant differences between the CK and SG groups in several carbon- and nitrogen-related categories (*Q* < 0.05). It is important to note that these predictions are computationally inferred from 16S rRNA gene amplicon data and should be regarded as functional hypotheses rather than direct measurements of in situ metabolic activity.

Within carbon cycling categories, the predicted abundances of chemoheterotrophy and aerobic chemoheterotrophy (CK: 42.73% ± 0.45% vs. SG: 34.03% ± 0.91%, Q = 0.011 (Table S7) and aerobic chemoheterotrophy (CK: 40.72% ± 0.46% vs. SG: 27.33% ± 2.19%, Q = 0.040) were significantly lower in the SG group (Table S7). In contrast, the predicted relative abundances of methylotrophy and its associated process, methanol oxidation, were significantly higher in the SG group (CK: 0.95% ± 0.08% vs. SG: 6.52% ± 1.26%, Q = 0.049) (Table S7). Additionally, chitinolysis, a function associated with the degradation of fungal cell walls, was significantly lower in the SG group (CK: 1.04% ± 0.15% vs. SG: 0.14% ± 0.01%, Q = 0.040) (Table S7).

Within nitrogen cycling categories, the predicted relative abundance of nitrogen fixation was significantly lower in the SG group (CK: 1.33% ± 0.06% vs. SG: 0.80% ± 0.08%, Q = 0.011) (Table S7), which coincided with the observed decrease in *Bradyrhizobium*, a genus containing known nitrogen-fixing members. Nitrate reduction was also significantly lower in the SG group (CK: 0.75% ± 0.11% vs. SG: 0.32% ± 0.02%, Q = 0.050) (Table S7). Conversely, the predicted relative abundance of ureolysis was significantly higher in the SG group (CK: 0.66% ± 0.18% vs. SG: 12.89% ± 1.92%, Q = 0.040) (Table S7). Furthermore, several additional predicted functions showed significant differences. Aromatic compound degradation (Q = 0.011) and predatory or exoparasitic activity (Q = 0.039) were significantly enriched in the SG group (Table S7). Denitrification-related functions were marginally lower in the SG group (Q = 0.053) (Table S7).

To further reveal the potential drivers of functional variation, Pearson correlation analysis was conducted between the top 20 most abundant genera at the genus level and the top 20 functional categories from FAPROTAX annotation (Figure 5B). The results showed that bacterial genera and functional categories clustered into two distinct modules corresponding to the CK and SG groups, respectively. Genera enriched in the CK group (e.g., *Bradyrhizobium*, *Steroidobacter*, *Streptomyces*) were positively correlated with functions including chemoheterotrophy, nitrogen fixation, and chitinolysis. In contrast, genera enriched in the SG group (e.g., *Methylotenera*, *Flavobacterium*, *Rhizobacter*) were positively correlated with methylotrophy, ureolysis and aromatic compound degradation. The concordance between taxonomic and functional variation indicated that changes in the abundance of corresponding functional taxa likely drove shifts in functional profiles.

## 4. Discussion

In this study, high-throughput sequencing of the 16S rRNA V4 region combined with FAPROTAX functional prediction was used to compare root-associated bacterial communities between short-term (CK, 4 mon) and long-term (SG, 16 mon) cultivated strawberry plants. As noted in the Methods, the SG group differed from the CK group simultaneously in soil cultivation history, plant age, and root developmental stage; therefore, all observed differences are interpreted as associations with cultivation duration rather than as effects of continuous cropping per season.

### 4.1. Cultivation Duration Is Associated with Shifts in Root-Associated Bacterial Community Structure and a Decline in Taxonomic Richness

Although the Shannon index, which reflects both richness and evenness and overall diversity, did not differ significantly between groups (Figure 1D), the number of observed ASVs was lower in the SG group (2144) than in the CK group (2506) (Figure 1C). The Shannon index did not change significantly despite a decline in observed ASVs, suggesting that the community may have lost some rare or low-abundance taxa without a major redistribution of dominance among the remaining groups. This decline is consistent with the reduced taxonomic richness observed under continuous cropping in tobacco [43], indicating that reduced microbial richness may be a common feature of long-term monoculture systems.

At the phylum level, the most notable change was a marked reduction in the relative abundance of Actinobacteria in the SG group (from 30.50% ± 3.62% to 12.85% ± 0.96%; *t*-test, *p* = 0.010), and LEfSe analysis identified Actinobacteria as a biomarker for CK (Figure 3A). Actinobacteria include many genera with documented biocontrol activity, such as *Streptomyces*, which can produce antifungal compounds, e.g., oligomycin [44], siderophores [45], and induce systemic resistance (ISR) in plants [46]. The sharp decline of this phylum in the SG group therefore suggests that the disease-suppressive capacity of the root-associated community may be compromised under long-term cultivation; however, confirmation at finer taxonomic resolution and with direct functional assays is required. Proteobacteria was the dominant phylum in both groups, with a moderately higher relative abundance in the SG group (74.27% ± 0.70%) than in the CK group (62.65% ± 5.13%; *p* = 0.044). Given the compositional nature of relative abundance data, this increase may partly reflect the passive consequence of the decline in Actinobacteria rather than absolute enrichment. Meanwhile, Bacteroidota was significantly enriched in the SG group (5.68% ± 1.39% vs. 1.64% ± 0.20%; *p* = 0.035) and was identified as its biomarker phylum (Figure 3A). Bacteroidota species possess strong abilities to degrade complex organic substrates [47]. Their enrichment may reflect increased availability of complex polysaccharides from accumulated plant debris or altered root exudate profiles under long-term cultivation [48], which could in turn influence bacterial community assembly.

At the genus level, the community composition shifted from *Streptomyces* (23.62% ± 2.78%), *Pseudomonas* (23.46% ± 4.66%), *Novosphingobium* (2.80% ± 0.08%)*,* and *Steroidobacter* (2.59% ± 0.17%) in CK to *unidentified Moraxellaceae* (13.10% ± 2.04%), *Pseudomonas* (7.73% ± 3.62%), *Flavobacterium*, *Rhizobacter* (12.26% ± 0.88%), and *Streptomyces* (9.09% ± 0.77%) in SG. The abundances of *Streptomyces* (from 23.6% to 9.1%, *p* = 0.008), *Steroidobacter* (from 2.6% to 0.6%, *p* = 0.01), *Bradyrhizobium* (from 0.9% to 0.3%, *p* = 0.0004), and *unidentified Rhizobiaceae* (from 1.4% to 0.8%, *p* = 0.005) were significantly reduced under continuous cropping (Figure 3B). *Bradyrhizobium* includes well-characterized nitrogen-fixing symbionts, and its decline is consistent with a potential reduction in biological nitrogen input, although direct measurements of nitrogen fixation activity would be necessary to confirm any functional impairment. The decline in *Streptomyces*, a genus known to produce diverse antimicrobial metabolites, is consistent with the hypothesis that long-term cultivation may be associated with a reduced reservoir of potentially beneficial taxa. In contrast, the enrichment of *Flavobacterium* and *Rhizobacter* in SG may reflect shifts in carbon metabolism and nutrient cycling. *Flavobacterium* can degrade diverse organic substrates and contribute to nutrient turnover [49,50]. *Rhizobacter* includes strains with varied ecological roles [51], and its specific functions under long-term cultivation warrant further strain-level investigation. In addition, the higher relative abundance of *Methylobacillus* in the SG group suggests that C1 compounds such as methanol may be more readily available in long-term cultivation systems [52].

In summary, cultivation duration was associated with a distinct root-associated bacterial community profile characterized by a marked decline in the relative abundance of Actinobacteria (including *Streptomyces*), enrichment of *Bacteroidota*, and reduced abundance of several genera with potential roles in nitrogen cycling and organic matter turnover. It should be reiterated that the present design cannot separate the effects of soil cultivation history from those of plant age. Therefore, the compositional differences reported here are best viewed as correlates of cultivation duration.

### 4.2. Cultivation Duration Is Associated with Altered Co-Occurrence Patterns Among Root-Associated Bacterial Genera

To further examine how continuous cropping influences the organization of root endophytic bacterial communities, a co-occurrence network was constructed using Pearson correlation coefficients. This network provides insight into how continuous cropping affects microbial interactions at the community level. It is important to note that with 3 composite replicates per group, the co-occurrence patterns presented here should be interpreted as exploratory. Correlation-based networks constructed from limited sample sizes are sensitive to outliers and may not reliably capture stable microbial associations. After filtering (|correlation coefficient| > 0.8), the network contained 26 genera with an average degree of 25.74 and a clustering coefficient of 0.73 (Table S3). A total of 3 genera (*Sphingobium* (Degree = 56), *Bradyrhizobium* (Degree = 54), and *Steroidobacter* degree = 52) exhibited the highest degree centrality, indicating that they were among the most highly connected genera in this network.

In network ecology, hub taxa are defined by their high degree of connectivity, serving as central nodes that facilitate resource exchange, metabolic coupling, and information flow across the community [53]. Notably, *Bradyrhizobium* and *Steroidobacter* were also significantly lower in abundance in the SG group (Figure 3B). The decline of these highly connected keystone taxa may impair the stability and functional coherence of the root-associated bacterial community. For instance, a study on root rot disease in Panax notoginseng revealed that following disease development, reduced negative connectivity among beneficial microorganisms disrupts the originally tightly connected network structure, diminishing the functional coherence of the entire microbial community and impairing the capacity to support plant health [54]. Similarly, research on apple trees also demonstrated significant differences in rhizosphere microbial community structure and functional profiles between visibly symptomatic diseased plants and asymptomatic counterparts, indicating that the key taxa maintaining network stability under healthy conditions undergo reorganization or decline during disease pathogenesis [55]. *Sphingobium* is known for its strong biodegradation capacity, particularly toward aromatic compounds such as phenolic acid autotoxins [56]. Its persistence as a highly connected genus in both groups may be related to the selective advantage conferred by this metabolic capability under conditions of phenolic acid accumulation.

*Bradyrhizobium*, as a nitrogen-fixing genus, likely reflects its involvement in nitrogen-cycling associations within the community. The lower relative abundance of this highly connected genus therefore suggests that long-term cultivation may be accompanied by changes in co-occurrence structure, potentially involving the weakening of certain highly connected nodes. *Steroidobacter* is associated with the degradation and transformation of complex organic compounds, including steroids [57]. Its reduced abundance may affect the decomposition of organic substrates in the rhizosphere and further alter nutrient cycling processes.

Overall, cultivation duration was associated with shifts in the relative abundance of key bacterial genera, and the lower abundance of highly connected genera such as *Bradyrhizobium* and *Steroidobacter* in the SG group suggests that network topology may differ between the two backgrounds. However, because correlation-based networks reflect statistical associations rather than direct ecological interactions, and because the sample size is limited, these results should be viewed as exploratory descriptions of co-occurrence patterns rather than as robust inferences of ecological interaction or stability. Future studies with greater replication and complementary experimental approaches (e.g., targeted co-culture assays or network analysis with independent datasets) will be needed to evaluate whether such co-occurrence patterns are reproducible and ecologically meaningful.

### 4.3. Cultivation Duration Is Associated with Differences in Predicted Functional Profiles of Root-Associated Bacterial Communities

FAPROTAX-based prediction (Figure 5) suggested differences in potential carbon- and nitrogen-related functional categories between the two groups of strawberry root-associated bacteria. It is essential to emphasize throughout this discussion that FAPROTAX infers functional potential from taxonomic composition of cultured representatives and does not directly measure in situ metabolic activity. Accordingly, the results should be regarded as functional hypotheses rather than confirmed metabolic differences.

To explore the taxonomic drivers underlying these predicted functional differences, Pearson correlation analysis was conducted between the top 20 most abundant genera and the top 20 functional categories from FAPROTAX annotation (Figure 5B). The results showed that bacterial taxa and functional categories clustered into two distinct modules corresponding to the CK and SG groups, respectively. Genera enriched in the CK group were positively correlated with core carbon and nitrogen cycling functions including chemoheterotrophy, nitrogen fixation and chitinolysis, whereas genera enriched in the SG group showed positive associations with methylotrophy, ureolysis and aromatic compound degradation. This consistent co-variation between taxonomic composition and functional potential indicates that the functional shifts under long-term cultivation are largely driven by the turnover of corresponding functional bacterial groups, rather than random functional fluctuations.

Within carbon-related categories, the predicted relative abundances of chemoheterotrophy (CK: 42.73% ± 0.45% vs. SG: 34.03% ± 0.91%, Q = 0.011) and aerobic chemoheterotrophy (CK: 40.72% ± 0.46% vs. SG: 27.33% ± 2.19%, Q = 0.040) were significantly lower in the SG group, by 8.7% and 13.4%, respectively. Chemoheterotrophic bacteria contribute to the degradation of organic matter and to carbon cycling in soil [58,59]. The lower predicted abundance of these categories in the SG group may reflect a shift in the predominant pathways of carbon utilization or a change in the availability of certain organic substrates; it should not be interpreted as indicating a decrease in total carbon availability in the root environment, since the primary carbon sources in the rhizosphere are root exudates and soil organic matter rather than microbial metabolism itself. In contrast, the predicted relative abundances of methylotrophy and methanol oxidation were significantly higher in the SG group (CK: 0.95% ± 0.08% vs. SG: 6.52% ± 1.26%, Q = 0.049). This enrichment suggests that long-term cultivation may favor taxa capable of utilizing C1 compounds, possibly reflecting changes in root exudate composition or in methanol accumulation. However, the present data are insufficient to establish a definitive metabolic shift toward C1 utilization. While *Methylobacillus* is a known methylotroph [60], *Flavobacterium* and *Rhizobacter* are metabolically versatile genera [61,62], and their enrichment alone does not provide conclusive evidence for a community-level transition to single-carbon metabolism. Direct measurements of methanol concentrations, exudate profiles, or transcriptional activity of C1 metabolism genes would be required to verify this inference. Such a potential shift could influence the forms and availability of carbon substrates in the root environment.

In nitrogen cycling, the predicted relative abundance of nitrogen fixation was significantly lower in the SG group (CK: 1.33% ± 0.06% vs. SG: 0.80% ± 0.08%, Q = 0.011), which coincided with the observed decrease in *Bradyrhizobium* (Figure 3B). While the direction of this change is consistent with a hypothesis of reduced nitrogen-fixation potential, the absolute magnitude of the difference (0.53 percentage points) is small, and FAPROTAX predictions do not match measured nitrogenase activity. Confirmation through nifH gene quantification or acetylene reduction assays would be necessary to determine whether biological nitrogen fixation capacity is meaningfully impaired. Nitrate reduction was also significantly lower in the SG group (CK: 0.75% ± 0.11% vs. SG: 0.32% ± 0.02%, Q = 0.050). Conversely, the predicted relative abundance of ureolysis was significantly higher in the SG group (CK: 0.66% ± 0.18% vs. SG: 12.89% ± 1.92%, Q = 0.040). Ureolysis converts urea to ammonia via urease, an important process in soil nitrogen transformation [63]. The enhanced ureolysis function may reflect an enrichment of urea-degrading bacteria under long-term cultivation, potentially associated with a greater reliance on organic nitrogen mineralization. However, without corresponding soil nitrogen measurements, the ecological significance of this predicted enrichment remains uncertain. Together, the lower predicted nitrogen fixation and the higher predicted ureolysis suggest a hypothetical shift in the predominant nitrogen-acquisition strategy from biological fixation to mineralization of organic nitrogen inputs. This hypothesis requires validation through direct functional assays and nitrogen budget measurements.

Several additional predicted functions showed significant differences between groups. Chitinolysis, a function associated with the degradation of fungal cell walls, was significantly lower in the SG group (CK: 1.04% ± 0.15% vs. SG: 0.14% ± 0.01%, Q = 0.040), which is consistent with the observed decline in Actinobacteria and may indicate a reduced potential for suppression of fungal pathogens. Aromatic compound degradation was significantly enriched in the SG group (Q = 0.011), potentially reflecting adaptation to the accumulation of phenolic autotoxins. Predatory or exoparasitic activity was also significantly higher in the SG group (Q = 0.039), suggesting altered trophic interactions under long-term cultivation.

This study has several limitations that should be acknowledged. First, the experiment used a single sampling time point and therefore could not capture temporal dynamics in bacterial community assembly or function. Second, each biological replicate consisted of composite roots from 3 plants, and each group included only 3 biological replicates, limiting statistical power and masking plant-to-plant variability. Moreover, sampling was restricted to thicker primary roots for consistency, potentially biasing community profiles toward taxa associated with mature root tissues. Second, the present network analysis was correlation-based and should therefore be interpreted as describing co-occurrence patterns rather than direct ecological interactions or community stability. Correlations are susceptible to random noise under small sample sizes, and the robustness of connectivity results is limited. Third, the FAPROTAX analysis provided prediction-based functional inference from 16S rRNA amplicon data rather than direct measurements of in situ metabolic activity. Fourth, although selected soil physicochemical properties were measured at the beginning of the experiment (see Section 2.1), these measurements were not repeated over time or at the time of sampling; therefore, the observed bacterial differences cannot be linked to specific soil-mediated mechanisms. Consequently, the diversity, network, and functional results presented here should be considered exploratory and hypothesis-generating. Future studies with redesigned sampling schemes, including multiple time points, surface-sterilized root compartments, increased replication, and complementary metagenomic or metatranscriptomic approaches, will be needed to robustly validate and mechanistically dissect the hypotheses generated here.

## 5. Conclusions

This observational study characterized the root-associated bacterial communities of strawberries under two cultivation regimes for short-term and long-term durations (4 and 16 mon, respectively). 16S rRNA gene amplicon sequencing revealed a distinct community composition between the two groups, with the long-term cultivation group showing a marked decline in the relative abundance of Actinobacteria (from 30.50% to 12.85%), particularly the genus *Streptomyces*, and a significant enrichment of Bacteroidota (from 1.64% to 5.68%). At the genus level, *Bradyrhizobium*, *Steroidobacter*, and *Streptomyces* were significantly depleted in the long-term group, while *Rhizobacter* and *Flavobacterium* were enriched. Co-occurrence network analysis identified *Sphingobium*, *Bradyrhizobium*, and *Steroidobacter* as the most highly connected genera, and the latter two were simultaneously reduced in relative abundance under long-term cultivation. FAPROTAX-based functional prediction suggested lower predicted chemoheterotrophy, aerobic chemoheterotrophy, nitrogen fixation, and chitinolysis, and higher predicted methylotrophy, methanol oxidation, and ureolysis in the long-term group; these predicted functional differences should be interpreted as hypotheses inferred from taxonomic composition rather than as direct measurements of metabolic activity.

These findings provide genus-level resolution of compositional and predicted functional shifts in the root-associated bacterial community during extended strawberry cultivation. The observed patterns, particularly the concurrent decline of Actinobacteria and *Bradyrhizobium*, partially align with microbial signatures reported in continuous cropping systems of other crops, suggesting that these taxa may represent candidate correlates of long-term cultivation stress. However, several limitations define the interpretive scope of this study. The single-time-point design, the confounding of cultivation duration with plant age, the limited replication (*n* = 3 composite replicates per group), the absence of root surface sterilization, and the lack of paired temporal soil data preclude causal attribution of the observed differences to continuous cropping within a season. Accordingly, the taxonomic and functional patterns reported here are best interpreted as exploratory associations with cultivation duration. Prospective studies incorporating age-controlled comparisons, longitudinal sampling, surface-sterilized root compartments, and direct functional measurements (e.g., metagenomics, nitrogenase activity assays) will be essential to validate and mechanistically dissect the candidate taxa and functional hypotheses generated by this work.

## Data Availability

The data that support the findings of this study have been deposited into CNSA (https://db.cngb.org/cnsa/, accessed on 20 January 2026) with accession number CNP0009395.

## Supplementary Materials

The following supporting information can be downloaded at: https://www.mdpi.com/article/10.3390/biology15151229/s1, Table S1: ASV statistical data of amplicon sequencing; Table S2: Detailed taxonomic information of amplicon sequencing; Table S3: Topological characteristic parameters of genus-level microbial communities in the co-occurrence network; Table S4: Sequencing quality metrics and alpha diversity indices of root-associated bacterial communities for each strawberry sample; Table S5: T-test statistical results of relative abundance of root-associated bacterial phylum between short-term (CK) and long-term (SG) strawberry planting groups; Table S6: T-test statistical results of relative abundance of root-associated bacterial genera between short-term (CK) and long-term (SG) strawberry planting groups; Table S7: T-test results of relative abundance of FAPROTAX predicted functional groups between short-term (CK) and long-term (SG) strawberry planting groups.
